# Molecular mechanism of enhanced ethanol tolerance associated with *hfq* overexpression in *Zymomonas mobilis*

**DOI:** 10.3389/fbioe.2022.1098021

**Published:** 2022-12-15

**Authors:** Ying Tang, Yi Wang, Qing Yang, Youpeng Zhang, Yalun Wu, Yongfu Yang, Meng Mei, Mingxiong He, Xia Wang, Shihui Yang

**Affiliations:** ^1^ State Key Laboratory of Biocatalysis and Enzyme Engineering, Environmental Microbial Technology Center of Hubei Province and School of Life Sciences, Hubei University, Wuhan, China; ^2^ Key Laboratory of Development and Application of Rural Renewable Energy, Biomass Energy Technology Research Centre, Biogas Institute of Ministry of Agriculture, Ministry of Agriculture, Chengdu, China

**Keywords:** *Zymomonas mobilis*, ethanol tolerance, Hfq, ROS-reactive oxygen species, protein-protein interaction (PPI), sulfate assimilation, cysteine biosynthesis, yeast endoplasmic reticulum sequestration screening system (YESS)

## Abstract

*Zymomonas mobilis* is a promising microorganism for industrial bioethanol production. However, ethanol produced during fermentation is toxic to *Z. mobilis* and affects its growth and bioethanol production. Although several reports demonstrated that the RNA-binding protein Hfq in *Z. mobilis* contributes to the tolerance against multiple lignocellulosic hydrolysate inhibitors, the role of Hfq on ethanol tolerance has not been investigated. In this study, *hfq* in *Z. mobilis* was either deleted or overexpressed and their effects on cell growth and ethanol tolerance were examined. Our results demonstrated that *hfq* overexpression improved ethanol tolerance of *Z. mobilis,* which is probably due to energy saving by downregulating flagellar biosynthesis and heat stress response proteins, as well as reducing the reactive oxygen species induced by ethanol stress *via* upregulating the sulfate assimilation and cysteine biosynthesis. To explore proteins potentially interacted with Hfq, the TEV protease mediated Yeast Endoplasmic Reticulum Sequestration Screening system (YESS) was established in *Z. mobilis*. YESS results suggested that Hfq may modulate the cytoplasmic heat shock response by interacting with the heat shock proteins DnaK and DnaJ to deal with the ethanol inhibition. This study thus not only revealed the underlying mechanism of enhanced ethanol tolerance by *hfq* overexpression, but also provided an alternative approach to investigate protein-protein interactions in *Z. mobilis*.

## Introduction

With the increasing demand for unsustainable fossil fuel reserves, the production of biofuels from renewable resources has become increasingly important and attracted considerable attentions such as bioethanol production by a variety of microorganisms, such as *Saccharomyces cerevisiae*, *Escherichia coli*, and *Zymomonas mobilis* by fermenting renewable resources such as lignocellulosic biomass.

*Z. mobilis* is a natural ethanologen exhibiting advantages of high sugar uptake, high specific ethanol productivity and yield, as well as high ethanol tolerance up to 16% (v/v) in batch fermentation (Rogers et al., 2007; Yang et al., 2013; Yang et al., 2016). Moreover, it does not require controlled oxygen addition during the fermentation process (He et al., 2014; Zhang K. et al., 2019). In addition, *Z. mobilis* has been genetically modified to enhance its robustness against different stresses including lignocellulosic hydrolysate inhibitors such as acetate, vanillin, and furfural (Yang Y. et al., 2018; Wang et al., 2018; Zhang K. et al., 2019). These advantages make *Z. mobilis* to be a promising microbial cell factory for industrial lignocellulosic bioethanol production.

However, the accumulation of ethanol produced during fermentation is still toxic to *Z. mobilis*, which is a bottleneck for bioethanol production improvement. Ethanol is a chaotropic compound that can promote changes in membrane composition and influence the structure and function of macromolecules such as proteins, nucleic acids, and lipids. At high concentrations, ethanol impedes the specific cell growth rate and viability of *Z. mobilis* cells and ultimately results in the death of the microorganism (Thanonkeo et al., 2007; Tan et al., 2016). To better understand and address these limitations, it is essential to obtain *Z. mobilis* mutant strains with improved ethanol tolerance.

Previous studies on molecular response to ethanol stress in *Z. mobilis* by transcriptomics and proteomics approaches demonstrated that multiple genes involved in different cellular processes were differentially regulated against ethanol stress such as membrane biogenesis, respiratory chain, DNA replication and recombination, transcriptional regulation, and general stress responses (He et al., 2012; Yang et al., 2013). Recently, Pallach *et al.* reported that one of the two cell surface exopolysaccharides in *Z. mobilis*, the galactose containing polymer (PS1), may have a crucial role in ethanol tolerance (Pallach et al., 2018). Another study in two ethanol-tolerant mutants exhibited that *clpP*, *spoT*/*relA*, and *clpB* genes seem to contribute to the ethanol tolerance in *Z. mobilis* (Carreon-Rodriguez et al., 2019).

Several global regulators including transcription factors were related to ethanol stress. For example, the heterologous expression of a global regulator gene *irrE* from *Deinococcus radiodurans* conferred high ethanol tolerance to *Z. mobilis* (Zhang et al., 2010). The random mutagenesis of gene encoding the native global transcription sigma factor (σ^70^, RpoD) *via* global transcription machinery engineering (gTME) enhanced ethanol resistance through modulating the transcriptional level (Tan et al., 2016). In addition, many efforts have also been focused on distinguishing the transcriptional regulators and transcription machinery, including small RNAs and RNA chaperones.

Hfq is a ubiquitous conserved Sm-like RNA-binding protein, which was originally identified as a host factor required for bacteriophage Qβ RNA replication in *E. coli*. Like eukaryotic and archaeal Sm/Lsm proteins, Hfq plays several roles in bacterial RNA metabolism, particularly to stabilize sRNAs (small non-coding RNAs) and promote their interactions with mRNAs leading to modulating stability and/or translation of these targets (Vogel and Luisi, 2011; Updegrove et al., 2016; Dos Santos et al., 2019). Beyond its sRNA-mediated regulation, Hfq was recently found to bind rRNA acting as a new ribosome biogenesis factor, and to bind tRNAs involved in the accuracy of protein synthesis. Moreover, Hfq is also capable to establish many protein-protein interactions (Yonekura et al., 2013; Dos Santos et al., 2019).

Given the central role played by Hfq in sRNA-mediated gene regulation in many bacteria, the protein has been widely recognized as a pleiotropic regulator of cell physiology, which particularly affects the cell response to multiple stresses. For example, at least three sRNAs, DsrA, RprA, and ArcZ interact with Hfq to positively regulate the *rpoS* transcript, which encodes the stress response sigma factor σ^S^ and controls >10% of all protein-coding genes in *E. coli* (Soper and Woodson, 2008; Frohlich and Gottesman, 2018).

Hfq in *Z. mobilis*, encoded by *ZMO0347*, was also discovered to contribute to multiple stress responses such as lignocellulosic hydrolysate inhibitor tolerance (Yang et al., 2010). Overexpression of *hfq* enabled the recombinant *Z. mobilis* an improved resistance to acetic acid, furfural and sugarcane bagasse hydrolysate compared to the parental strain (Nouri et al., 2020). Moreover, the 5′ untranslated regions (5′ UTRs) of *hfq* gene as the regulatory element was revealed to downregulate downstream gene expression under ethanol stress in *Z. mobilis* (Cho et al., 2017). These findings suggested an important role of Hfq in dealing with ethanol stress in *Z. mobilis*. However, the full repertoire of Hfq-dependent gene regulation response to ethanol has not been elucidated, although omics efforts have been carried out to understand ethanol stress responses in *Z. mobilis* (Zhang et al., 2010; Tan et al., 2016; Han et al., 2020). In this study, the role of *hfq* on ethanol tolerance in *Z. mobilis* was investigated by constructing mutant strains of *hfq* deletion or overexpression, and their molecular responses were characterized using genetic approaches and transcriptomic study.

## Materials and methods

### Strains, media, and growth conditions

Bacterial strains and plasmids used in this study are listed in Supplementary Table S1. *Z. mobilis* ZM4 (ATCC 31821) was used as the parental strain in this study. Generally, ZM4 and its derivative strains are cultured at 30°C with shaking at 100 rpm in RM medium (50 g/L glucose, 10 g/L yeast extract, 2 g/L KH_2_PO_4_, and 1.5% agar for solid). To avoid the effect of other amino acid nutrients in RM medium, MM medium (50 g/L glucose, 1 g/L KH_2_PO_4_, 1 g/L K_2_HPO_4_, 1 g/L (NH_4_)_2_SO_4_, 0.5 g/L NaCl, 0.42 g/L MgCl_2_ 6H_2_O, 0.001 g/L calcium pantothenate, and 1.5% agar for solid) was used in this study for Na_2_SO_4_ supplementation experiments, and 4 g/L Na_2_SO_4_ was added. The final concentration of exogenous ethanol added to the RM and MM medium is 8% and 3% (v/v), respectively.

*E. coli* DH5α was used for plasmid construction in this study. All *E. coli* strains were cultured in LB medium (10 g/L tryptone, 5 g/L yeast extract, 10 g/L NaCl, and 1.5% agar for solid) at 37°C, 250 rpm. When required, 100 and 200 μg/ml spectinomycin was added for *E. coli* or *Z. mobilis*, respectively.

### Genetic manipulation and recombinant strain construction

The shuttle plasmid pEZ15A was used for *hfq* overexpression in *Z. mobilis* ZM4 with its native promoter and the recombinant strain named ZM4-*hfq*. The plasmid pL2R was used for *hfq* deletion using the native Type I-F CRISPR-Cas system of *Z. mobilis* (Zheng et al., 2019). Plasmid pEZ15A was transformed into the wild type and the *hfq* deletion strain, which named ZM4 and ZM4-Δ*hfq*, respectively.

For the gene-deficient mutant construction, interference plasmids were initially constructed with spacer. The spacer was designed to bear the entire 32-bp sequences containing a 5′-CCC-3′ PAM. The editing plasmid pL2R was digested with *Bsa* I at 37°C overnight. Oligonucleotides were annealed by first heating the reaction mixture to 95°C for 5 min and subsequently cooling down gradually to room temperature. The annealed spacer and the digested linear DNA vector were enzymatically linked with T4 ligase at 18°C overnight, and then transformed into DH5α competent cells to generate genome engineering plasmids.

For all plasmid construction, primers were designed to contain a region of 15–25 nucleotides that overlap with adjacent DNA fragments and synthesized by TsingKe (Beijing, China) (Supplementary Table S2). All plasmids were assembled by Gibson assembly method (Gibson et al., 2009). Gene and vector fragments amplified by primer pairs were purified and then ligated through the T5 exonuclease (NEB, WA, United States). The gene fragment was mixed with the vector at a 3:1 M ratio, 0.5 U T5 exonuclease (NEB, MA, United States) and 0.5 μl buffer 4 (NEB, MA, United States) were then added, with ddH_2_O added to the final volume of 5 μl. All reagents were mixed and incubated on ice for 5 min before being added to chemically competent *E. coli* cells. After ice incubation for 30 min and heat-shock for 45 s at 42°C, held it on ice for 2 min before 100 μl NZY medium (5.0 g/L yeast extract, 5 g/L NaCl, 1.2 g/L MgCl_2_, 1.5 g/L MgSO_4_, 3.6 g/L glucose, 10 g/L casein enzymatic hydrolysate NZ amine®) were added to the mixture above, and recovered at 37°C, 250 rpm. The cells were plated on LB agar plates containing 100 μg/ml spectinomycin, the recombinant was screened by colony PCR and confirmed by Sanger Sequencing (TsingKe Biotechnology, Beijing, China).

After identification, the recombinant plasmid was transformed into *Z. mobilis* competent cells *via* electroporation (0.1-cm electrode gap, 1600 V, 200 Ω, 25 μF) using a Gene Pulser® (Bio-Rad, CA, United States) following the method developed for *Z. mobilis* (Okamoto and Nakamura, 1992). The correct colonies were selected by colony PCR. After three to five generations purification on RM agar plates with 200 μg/ml spectinomycin supplementation, the correct recombinant was stored at −80°C.

### Cell growth and fermentation analysis

Seed culture of *Z. mobilis* was prepared by reviving the frozen glycerol stocks in 50 ml flasks containing 40 ml RM medium. After culturing overnight without shaking to the mid-exponential phase, the seed culture was harvested and inoculated in 50 ml shake flasks containing 40 ml RM or MM medium with an initial OD_600 nm_ value of 0.1. During the fermentation, cell growth in terms of the absorbance value (OD_600_) was measured at 600 nm by a spectrophotometer (UV-1800, AOE, China) at different time points. Samples were centrifuged at 12,000 rpm for 2 min, collected supernatants were passed through 0.22-μm filters and stored at −80°C for subsequent HPLC analysis if needed. Glucose and ethanol in the culture supernatant were determined using a HPLC system LC-20AD (Shimadzu, Japan) with an Aminex HPX-87H column (300 × 7.8 mm, Bio-Rad, CA, United States) at 65°C. Elution was performed with 5 mM H_2_SO_4_ at 0.5 ml/min.

### RNA-seq and statistical analyses

Cell culture samples in different conditions were collected at the mid-exponential phase. According to standard Illumina protocols, RNA-Seq was carried out by GENEWIZ (Suzhou, China) with a library construction kit (NEBNext® Ultra™ Directional RNA Library Prep Kit for Illumina®) and an Illumina HiSeq 2000 instrument. After RNA-seq fastq data quality was evaluated by FastQC software (Babraham Bioinformatics, United Kingdom). Data were filtered and those passed the quality control were imported to the CLC Genomics Workbench (version 14.0) for reads trimming and RNA-Seq analysis to get the RPKM value (reads mapping to the genome per kilobase of transcript per million reads sequenced) of each gene. Genome sequence of *Z. mobilis* was used as the reference for RPKM calculation (Yang S. et al., 2018).

Gene expression normalization, analysis of variance (ANOVA), and hierarchical clustering analysis were conducted using JMP Genomics (Ver. 9.0, SAS Inc., NC, United States) to identify differentially expressed genes at different conditions. Significantly differentially expressed genes were identified with *p*-value ≤0.05 and log_2_-fold change ≥1 (significant induction) or ≤−1 (significant inhibition) as selection thresholds. The one-way ANOVA analysis was performed by JMP Genomics and only those with *p*-value ≤0.05 were considered for further analysis.

### Measurement of intracellular ROS

Intracellular ROS levels were measured by 2′,7′-dichlorodihydro-fluorescein diacetate (H_2_DCF-DA) (Beyotime Biotechnology, Hangzhou, China) (Yan et al., 2022). Cells were collected at the mid-log phase (total OD_600_ was 1.0) at 12,000 rpm for 1 min, washed with phosphate buffer saline (PBS) and then resuspended in 500 μl PBS with the final concentration of H_2_DCF-DA at 100 μM. After incubating in darkness for 1 h at 30°C, 100 rpm, cells were collected, washed three times with PBS and resuspended in 300 μl PBS. The CytoFLEX FCM flow cytometry (Beckman Coulter, CA, United States) was used to detect the DCF fluorescence with excitation and emission wavelength of 488 nm and 525 nm, respectively. Cells with fluorescence intensities ranging 10^3^–10^5^ were selected and counted, and 20,000 cells were analyzed for each sample.

### Yeast endoplasmic reticulum sequestration screening system

The plasmid pESE which is modified from pESD (Yi et al., 2013) was used and the TEV protease mediated Yeast Endoplasmic Reticulum Sequestration Screening (YESS) system was construction in *Z. mobilis* for the Protein-Protein interaction detection (Supplementary Figure S1). For the recombinant plasmid construction, two target genes and the vector fragment were PCR amplified and purified, and then assembled by the Golden gate assembly strategy. The reaction included 0.05 mol gene fragments, 20 ng pESE, 1 μl T4 ligase buffer (Thermo), 0.2 μl T4 ligase (Thermo), 0.3 μl *Bsa* I (NEB), 0.1 μl BSA (Takara), and with ddH_2_O added to a final volume of 10 μl. All reagents were mixed and incubated on 37°C for 3 h before being added to chemically competent *E. coli* cells. After being incubated on ice for 30 min, heat-shocked for 45 s at 42°C, and held on ice for 2 min, 100 μl of NZY medium was added into above mixture and recovered for at least 1 h at 37°C with shaking at 250 rpm. The cells were plated on LB agar plates containing 50 μg/ml ampicillin, the recombinants were then screened by colony PCR and confirmed by Sanger Sequencing (TsingKe Biotechnology, Beijing, China).

The correct recombinant plasmids were transformed to *S. cerevisiae* EBY100 competent cells and then plated on SD-UT agar plates (2% galactose, 0.67% yeast nitrogen base w/o amino acids, 1% casamino acids, 1.5% agar) at 30°C. Single colony was selected and cultivated in 25 ml tube containing 5 ml SD-UT medium for 14 h with shaking at 200 rpm. The seed culture was then harvested and transferred into 25 ml tube containing 5 ml SD-DT medium (2% glucose, 0.67% yeast nitrogen base w/o amino acids, 1% casamino acids, 1.5% agar) with an initial OD_600 nm_ value of 0.8. After induction at 30°C for 8 h, 10^6^ cells were collected by centrifugation, washing two times with 150 μl PBS buffer at 3,000 rpm, 4°C for 1 min, and finally resuspended in 20 μl PBS. For Fab display analysis, cells were incubated with 0.1 μl anti-HA-FITC and/or 0.1 μl anti-FLAG-iFlor647 (GenScript, Nanjing, China) at 4°C for 15 min in darkness. After washed three times with PBS (pH 7.4) supplemented with 0.5% BSA at 3,000 rpm, 4°C for 1 min, the sample was resuspended in 300 μl PBS (pH 7.4) with 0.5% BSA. Flow cytometry analysis was performed by the Beckman Coulter CytoFLEX (Brea, CA) equipped with 488 and 633 nm lasers and 525/40 and 660/20 nm band-pass filters.

## Results and discussion

### *Hfq* overexpression enhanced ethanol tolerance in *Z. mobilis*

**FIGURE 1:**
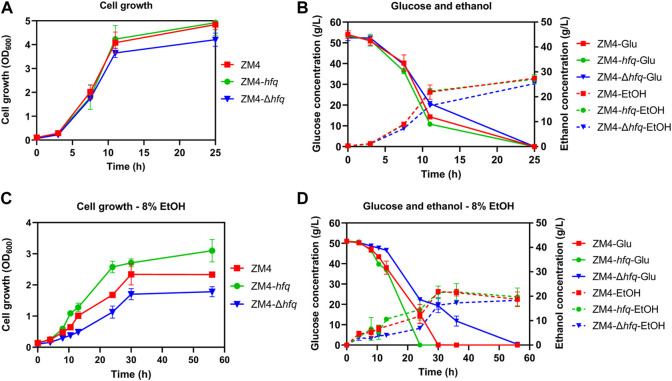
The cell growth, glucose consumption and ethanol production of *Z. mobilis* strains in the absence (**A,B**) or presence of 8% (v/v) ethanol (**C,D**). *Z. mobilis* wild-type (ZM4), *hfq* overexpression (ZM4-*hfq*). *hfq* deletion (ZM4-Δ*hfq*) strains were cultured in RM medium at 30°C. The error bars indicate standard deviations based on three replicates. Glu: glucose, EtOH: ethanol produced from glucose with the exogenous ethanol supplemented into the medium subtracted from the total ethanol amount in the medium.

To investigate the effect of *hfq* on ethanol tolerance in *Z. mobilis*, recombinant strains with *hfq* overexpression (ZM4-*hfq*) or *hfq* deletion (ZM4-Δ*hfq*) were constructed and evaluated in terms of cell growth, sugar utilization, and ethanol production. As shown in Figure 1A, the *hfq* overexpression strain ZM4-*hfq* grew normally without ethanol treatment and achieved a final OD_600_ value of 4.92 that was similar to the wild-type with OD_600_ value of 4.84. While, the *hfq* deletion strain ZM4-Δ*hfq* grew slightly slower than wild type with a final OD_600_ value of 4.20. Corresponding to the slower cell growth, ZM4-Δ*hfq* exhibited slower glucose consumption and ethanol production than the control and ZM4-*hfq* (Figure 1B). These results indicated that Hfq plays roles for normal cell growth even without ethanol stress inhibition.

Fermentation performance was further evaluated with 8% exogenous ethanol added into the media. Cell growth of ZM4 was effectively improved with *hfq* overexpression. ZM4-*hfq* grew into the stationary phase around 24 h and achieved a final OD_600_ value of 3.10, while wild-type strain had an OD_600_ value of 2.33 only after 56 h (Figure 1C). Consistently, *hfq* deletion in *Z. mobilis* significantly decreased the ethanol tolerance. The maximum biomass of strain ZM4-Δ*hfq* exposure to 8% ethanol was quite lower than that of the control with OD_600_ of 1.78 vs. 2.33. Corresponding to the cell growth, ZM4-*hfq* strain accelerated the glucose consumption and ethanol production, while strain ZM4-Δ*hfq* was in reverse. As shown in Figure 1D, all glucose was consumed by ZM4-*hfq* to achieve a maximum ethanol titer of 25.82 g/L within 24 h, almost 6 h faster than that of control, while 6.83 g/L residual glucose was still detected at 36 h by strain ZM4-Δ*hfq*. All these results indicated that *hfq* in *Z. mobilis* contributes to the ethanol tolerance, just as its positive roles responding to other stresses such as furfural and acetic acid (Yang et al., 2010; Nouri et al., 2020).

### Multiple genes regulated in the *hfq* recombinant strains under ethanol stress

Previous reports have shown that Hfq is associated with the regulation of numerous cellular pathways (Vogel and Luisi, 2011; Sharma et al., 2018; Han et al., 2019). To gain insights into the genes regulated by Hfq, and to illustrate the molecular mechanism of the positive role of Hfq overexpression on ethanol tolerance in *Z. mobilis*, RNA-Seq was employed to explore the global transcriptional differences of ZM4 and its *hfq* derivatives ZM4-*hfq* and ZM4-Δ*hfq* under different conditions of E0 (control, without exogenous ethanol treatment) and E8 (8% exogenous ethanol treatment) (Figure 1). Differentially expressed genes (DEGs) between different strains across different growth conditions were analyzed through analysis of variance (ANOVA) with a total of 628 DEGs identified (Supplementary Tables S3, S4).

The expression of *hfq* gene was examined first. As expected, the transcript of *hfq* was not detected in ZM4-Δ*hfq*, while the expression of *hfq* in ZM4-*hfq* had a 3.2-fold upregulation compared with the wild-type ZM4 (*p*-value ≤0.05) (Supplementary Tables S3, S4). The gene expression of *hfq* confirmed that recombinant strains ZM4-Δ*hfq* and ZM4-*hfq* were both constructed successfully, and the global gene expression was then further analyzed.

Consistent with previous studies (He et al., 2012; Yang et al., 2013), the presence of ethanol made many genes differentially expressed (E8 vs. E0) including 92 upregulated and 61 downregulated genes, which were mainly associated with cell wall/membrane biogenesis, metabolism, transcription, and general stress response. The effect of ethanol stress on three strains ZM4, ZM4-*hfq*, and ZM4-Δ*hfq* were further compared and analyzed. The results showed that exogenous ethanol stress regulated more than 200 genes in all three strains (Supplementary Table S3). This result suggested that despite of *hfq* overexpression, *Z. mobilis* remained challenged by the ethanol stress, which was consistent with the decreased cell growth and reduced biomass accumulation of ZM4-*hfq* in the presence of 8% ethanol compared with that without ethanol treatment (Figures 1A, C).

The detailed gene expression information of different strain comparisons was analyzed as well (Supplementary Table S4). Unlike the dramatic transcriptomic changes in response to ethanol stress, less differentially expressed genes were observed among strains. Under E0 condition without exogenous ethanol treatment, 58 genes were differentially expressed in ZM4-Δ*hfq* compared with ZM4, and 143 genes were differentially expressed when compared with ZM4-*hfq.* Meanwhile, only 30 genes were detected to be differentially regulated in ZM4-*hfq* compared with ZM4. When 8% exogenous ethanol was supplemented into RM (E8 condition), more differentially expressed genes were identified. Eighty genes were differentially expressed in ZM4-*hfq* compared with ZM4, while 77 genes and 181 genes were differentially expressed in ZM4-Δ*hfq* compared with ZM4 and ZM4-*hfq,* respectively. The result of more genes differentially expressed under E8 condition was consistent with the fermentation performance analyses that *hfq* recombinant strains were obviously different from the parental strain ZM4 under ethanol stress conditions (Figure 1). All these genes identified between strain comparisons could be closely associated with the positive role of Hfq on ethanol tolerance in *Z. mobilis*.

### Hfq negatively regulated flagellar proteins in *Z. mobilis*

Bacterial flagellum is a complex and dynamic nanomachine appended on the cell body that provides motility (Soutourina and Bertin, 2003). Recent studies reported that these complex organelles also play an important role in bacterial survival, reproduction, and pathogenicity, such as adhesion to a variety of substrates, secretion of virulence factors, and formation of biofilms (Nedeljkovic et al., 2021). Gene expression without ethanol supplementation (E0) showed that 20 flagellar related genes (total 34 genes in *Z. mobilis*), including *flgBCEGHIJKL*, *flhA*, *fliCEFGHIKL*, and *motAB* encoding flagellar structure proteins, motor proteins and biosynthesis proteins were upregulated in ZM4-Δ*hfq* compared with the wild-type ZM4 or ZM4-*hfq*, and most of these genes were upregulated as well in the presence of 8% ethanol (E8) (Table 1, Supplementary Table S4).

**TABLE 1:** List of significantly differentially expressed genes in different functional categories between strain comparisons under 8% (v/v) ethanol treatment.

Locus ID	Gene	Product	ZM4-Δ*hfq* vs. ZM4	ZM4-*hfq* vs. ZM4	ZM4-Δ*hfq* vs. ZM4-*hfq*
Flagellar proteins					
ZMO0602	*motB1*	Flagellar motor protein Motb	—	—	—
ZMO0603	*motA1*	Flagellar motor protein Mota	—	—	—
ZMO0604	*flgL*	Flagellin domain protein	—	—	1.21
ZMO0605	*flgK*	Flagellar hook-associated protein FlgK	—	—	1.11
ZMO0606	-	Flagellar rod assembly protein FlgJ	1.34	—	1.79
ZMO0607	*flgI*	Flagellar P-ring protein	—	—	1.13
ZMO0608	*flgH*	Flagellar L-ring protein	—	—	1.08
ZMO0609	*flgG*	Flagellar basal-body rod protein FlgG	—	—	1.05
ZMO0613	*flgC*	Flagellar basal-body rod protein FlgC	—	—	1.08
ZMO0614	*flgB*	Flagellar basal-body rod protein FlgB	1.32	—	—
ZMO0629	*fliC*	Flagellin domain-containing protein	2.19	—	2.47
ZMO0631	-	Fis family sigma54-specific regulator	1.28	—	1.34
ZMO0632	*fliE*	Flagellar hook-basal body complex subunit FliE	1.49	—	2.10
ZMO0634	*fliF*	Flagellar M-ring protein FliF	1.24	—	2.06
ZMO0635	*fliG*	Flagellar motor switch protein FliG	1.46	—	2.06
ZMO0636	*fliH*	Negative regulator of FliI ATPase	—	−1.03	1.90
ZMO0637	*fliI*	Flagellum-specific ATP synthase FliI	—	—	1.34
Sulfur metabolism					
ZMO0003	*cysC*	Adenylyl-sulfate kinase	—	—	−2.21
ZMO0004	*cysN*	Sulfate adenylyltransferase large subunit	—	2.11	−2.70
ZMO0005	*cysD*	Sulfate adenylyltransferase small subunit	—	1.92	−2.68
ZMO0007	*cysH*	Phosphoadenosine phosphosulfate reductase	—	1.59	−1.73
ZMO0008	*cysI*	Sulfite reductase hemoprotein beta-component	—	1.64	−2.03
ZMO0009	*cysJ*	Sulfite reductase flavoprotein alpha chain	—	—	−1.89
ZMO1000	*metE*	5-methyltetrahydropteroyltriglutamate--homocysteine S-methyltransferase	—	2.12	−2.56
ZMO1684	*serC*	Phosphoserine aminotransferase	—	1.09	—1.28
ZMO1685	*serA1*	D-3-phosphoglycerate dehydrogenase	—	1.55	—1.83
Heat shock response					
ZMO0016	*grpE*	GrpE protein	—	−1.10	1.06
ZMO0246	*hslV*	ATP-dependent protease subunit HslV	—	−2.06	2.21
ZMO0247	*hslU*	Heat shock protein atpase subunit HslU	—	−1.77	1.76
ZMO0376	*lon1*	Endopeptidase La	—	−1.21	1.62
ZMO0405	*clpA*	ATP-dependent Clp protease subunit ClpA	—	—	1.62
ZMO0660	*dnaK*	Chaperone protein DnaK	—	−1.51	1.60
ZMO0661	*dnaJ*	Chaperone protein DnaJ	—	−2.08	1.65
ZMO0948	*clpP*	ATP-dependent Clp protease subunit ClpP	—	—	1.27
ZMO0949	*clpX*	ATP-dependent Clp protease subunit ClpX	—	—	1.17
ZMO0989	*ibpA*	Heat-shock protein IbpA	—	−2.96	3.06
ZMO1424	*clpB*	ATP-dependent chaperone ClpB	—	−2.05	2.53
ZMO1704	*lon2*	Protease La (LON) substrate-binding domain	—	−1.65	2.22
ZMO1928	*groES*	Chaperonin Cpn10	—	−2.06	2.39
ZMO1929	*groEL*	Chaperonin GroEL	—	−2.20	2.28

a, gene expression represented the log_2_-fold changes; —for not differentially expressed; red or blue for up-regulated or down-regulated, respectively.

Moreover, the expression of *fliA*, which encodes an alternative sigma factor specific for the flagellar regulons including *flgK* and *fliC* (Ohnishi et al., 1990), was two folds upregulated in ZM4-Δ*hfq* compared with the wild-type ZM4 with or without ethanol presence. These results collectively suggested that Hfq in *Z. mobilis* might constitutively mediate through the sigma factor FliA to negatively regulate the expression of flagellar proteins, which has been reported in other Gram-negative microbes, such as *Cronobacter sakazakii* (Kim et al., 2015). Previous transcriptomic analyses in *Z. mobilis* reported that flagellar related genes were downregulated to help conserve energy from cell motility for survival in the stressful conditions (He et al., 2012; Yang et al., 2013). Therefore, the less ethanol tolerance in ZM4-Δ*hfq* might be attributed to the limited energy resulting from the upregulation of the energy-costly flagellar assembly process without the negative regulation of Hfq.

In addition, gene expression in response to ethanol stress showed that above flagellar related genes were downregulated in all three strains, especially in ZM4-Δ*hfq* with more than 2-fold repression (Supplementary Table S3). The downregulation of flagellar proteins in ZM4-Δ*hfq* indicated that other mechanisms of flagellar regulation may exist in *Z. mobilis* especially under the ethanol stress condition, and further study is needed to unravel the underlying mechanisms. Altogether, the gene expression data suggested that the negative regulation of Hfq on these flagellar related genes is beneficial but may not be sufficient for the ethanol tolerance in *Z. mobilis.*

### Hfq mediated sulfur metabolism to eliminate the oxidative stress induced by ethanol stress

**FIGURE 2:**
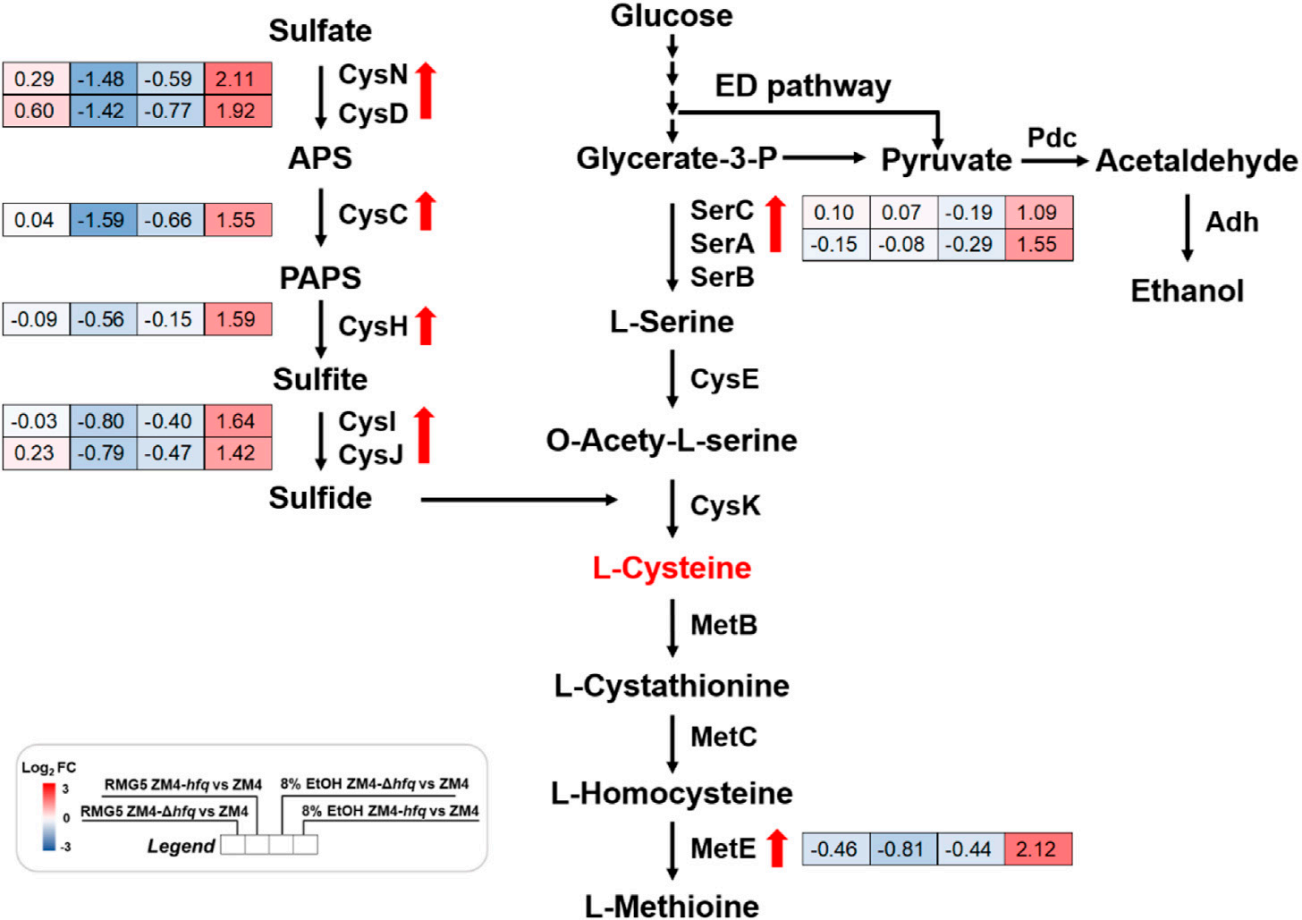
Transcriptional changes of genes involved in the sulfate assimilation and cysteine biosynthesis in *Z. mobilis* cultured in different conditions. Red and blue in the gene expression represent up-regulation and down-regulation respectively. Red arrows indicate the genes were significantly upregulated in ZM4-*hfq* compared with ZM4. APS: adenosine 5′-phosphosulfate, PAPS: 3′-phosphoadenylyl sulfate.

A gene cluster involved in sulfate assimilation and cysteine biosynthesis pathway was identified by comparing gene expression among strains under ethanol stress. Gene expression under ethanol treatment (E8) showed that all six genes associate with sulfate assimilation, *cysCND* (*ZMO0003*-*0005*) and *cysHIJ* (*ZMO0007*-*0009*), were upregulated in ZM4-*hfq* compared with ZM4-Δ*hfq* or ZM4. Similar upregulation of gene expression in ZM4-*hfq* was observed in other genes involved in cysteine biosynthesis, including *serA1* (*ZMO1685*) and *serC* (*ZMO1684*) (Table 1; Figure 2, Supplementary Table S4). However, such upregulated gene cluster was not induced under the condition without ethanol stress (E0). These results indicated that the sulfur metabolism including the cysteine biosynthesis was induced under the ethanol stress condition by *hfq* overexpression in *Z. mobilis*. Previous study confirmed that cysteine supplementation enhanced the tolerance in *Z. mobilis* to different inhibitors including ethanol (Yan et al., 2022). Therefore, *hfq* overexpression in ZM4-*hfq* might regulate the sulfate assimilation and cysteine biosynthesis for enhanced ethanol tolerance.

**FIGURE 3:**
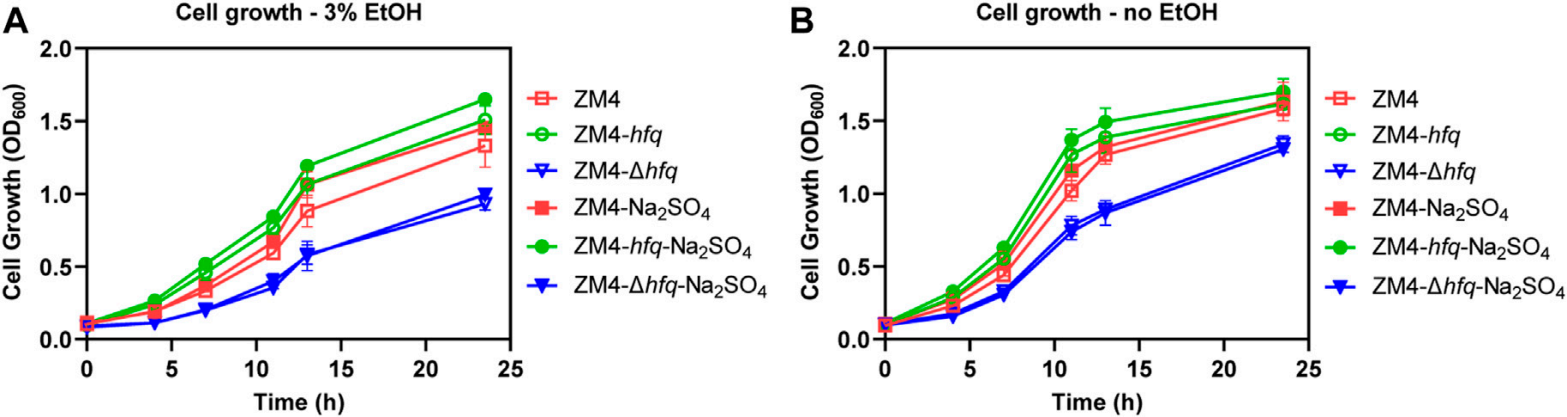
Effects of Na_2_SO_4_ addition on cell growth of *hfq* recombinant strains and wild type. 4 g/L Na_2_SO_4_ was added in the MM media and cell growths of *Z. mobilis hfq* recombinant strains were compared with the wild-type strain with **(A)** or without **(B)** 3% (v/v) ethanol treatment. EtOH represent ethanol.

To evaluate the hypothesis that *hfq* overexpression enhanced sulfur metabolism and therefore played a protective role against the ethanol stress, 4 g/L Na_2_SO_4_ as the substrate for sulfate assimilation was supplemented into the medium and the cell growths of ZM4 and its *hfq* recombinant strains were detected (Figure 3). To avoid the effect of other sulfur-containing nutrients in RM medium, MM medium was used (Yan et al., 2022). Consistent with the result in rich medium (Figure 1), *hfq* overexpression strain ZM4-*hfq* exhibited an enhanced tolerance to 3% ethanol in MM media and achieved a final OD_600_ of 1.51, clearly higher than the wild-type with OD_600_ of 1.33. While, the *hfq* deletion strain ZM4-Δ*hfq* had the lowest cell growth with the final OD_600_ of 0.93 among three strains (Figure 3A). When Na_2_SO_4_ was added in the media, the cell growth of ZM4-*hfq* under ethanol treatment was further improved to a final OD_600_ of 1.65, while no influence was observed for *hfq* deletion strain ZM4-Δ*hfq*.

Moreover, an improvement responding to ethanol stress was detected as well in the wild-type ZM4 with the Na_2_SO_4_ supplementation, which suggested that the native gene expression of *hfq* in ZM4 would effectively regulate the sulfate assimilation process with Na_2_SO_4_ as the substrate. Since that MM medium is regarded as a stressful environment for *Z. mobilis* (Yang et al., 2014), Na_2_SO_4_ supplementation also slightly improved the cell growth of ZM4-*hfq* and ZM4 without ethanol treatment (Figure 3B). However, there is still no improvement observed for ZM4-Δ*hfq*. Taken together, Na_2_SO_4_ supplementation results suggested that the *hfq* overexpression in ZM4-*hfq* positively regulated the sulfate assimilation process, and Na_2_SO_4_ could be assimilated to deal with the stressful condition.

Previous studies in *E*. *coli*, *S*. *cerevisiae*, and *Z. mobilis* demonstrated that the sulfate assimilation was induced in response to stresses (Miller et al., 2009; Kanna and Matsumura, 2015; Zhang Y. et al., 2019). Recent study in *Z. mobilis* 8b further confirmed that cysteine supplementation in the growth media boosted glutathione synthesis or H_2_S release effectively leading to the reduced accumulation of reactive oxygen species (ROS) induced by inhibitor stress (Yan et al., 2022). To evaluate the impact of upregulated sulfur metabolism on ROS for ethanol tolerance improvement in ZM4-*hfq*, the cellular ROS levels in *hfq* recombinant strains under ethanol treatment were further examined.

**FIGURE 4:**
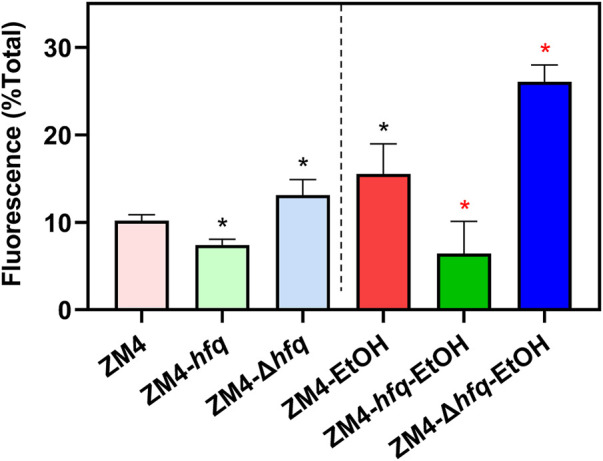
The effect of Hfq overexpression on reactive oxygen species (ROS) accumulation induced by ethanol in *Z. mobilis*. *Z. mobilis* strains were cultured in MM media with or without 3% (v/v) ethanol treatment, and EtOH represents the ethanol treatment. One-way ANOVA analysis was conducted for ROS detection with ZM4 (black asterisk, *p*-value <0.05) or ZM4-EtOH (red asterisk, *p*-value <0.05) condition as the control.

As shown in Figure 4, the intracellular ROS accumulation was detected in the wild-type ZM4 with ethanol treatment (10.19%–15.53%, *p*-value <0.05). Corresponding to the cell growth performance responding to ethanol stress (Figure 3), the intracellular ROS level was decreased by *hfq* overexpression in ZM4-*hfq* compared with the wild-type control ZM4 (15.53%–6.42%, *p*-value <0.05), while increased by *hfq* deletion in ZM4-Δ*hfq* (15.53%–26.07%, *p*-value <0.05). Similar alterations were detected in strain comparisons without ethanol presence (Figure 4). Collectively, the results suggested that *hfq* overexpression in *Z. mobilis* was involved in the positive regulation of sulfate assimilation and cysteine biosynthesis, and thus contributed to the effective elimination of the ROS induced by ethanol inhibition and finally enhanced the ethanol tolerance of *Z. mobilis.*

The major stress sigma factor RpoS of *E. coli* and *Salmonella* is the master regulator of oxidative stress response, and depends on Hfq for translation (Frohlich and Gottesman, 2018). *Z. mobilis* does not possess a *rpoS* gene, but its genome contains other five sigma factors genes. RpoE (sigma E) in *Z. mobilis* potentially is a RpoS-like regulatory factor, and plays a critical role in various stress conditions including ethanol stress responses (Seo et al., 2005). Recent study in the facultative phototrophic bacterium *Rhodobacter sphaeroides* demonstrated that sigma factor RpoE is involved in a specific photo-oxidative stress response, and sRNA RSs0019 controlled by the sigma factor RpoE contributes to the balance of sulfate uptake and biosynthesis of sulfur-containing amino acids to guarantee appropriate cysteine and glutathione levels under the given environmental conditions (Hess et al., 2014). These evidences suggested a potential role of *rpoE* in the regulation of sulfur metabolism against the oxidative stress in *Z. mobilis*.

In this study, the gene expression of *rpoE* (*ZMO1404*) was not influenced in both *hfq* recombinant strains. However, Hfq may only influence the RpoE activity without affecting its protein levels just as observed in *R*. *sphaeroides* (Berghoff et al., 2011). In addition, recent study in *Z. mobilis* identified two sRNAs, Zms4 and Zms6, that might affect a wide range of sRNAs through modulating *hfq* expression indirectly and specifically co-regulate some pathways important to ethanol stress, including sulfate assimilation and cysteine biosynthetic processes (Han et al., 2020). All these results support the role of Hfq on sulfur metabolism in response to ethanol stress. However, considering the versatile function of Hfq (Dos Santos et al., 2019), further studies are necessary to define the regulation of Hfq in *Z. mobilis*.

### Hfq involved in modulating RpoH-mediated cytoplasmic heat shock response

*Z. mobilis* can regulate general stress-response genes especially those in heat shock response to protect proteins from the damage caused by the stressful environments (Yang et al., 2014; Yi et al., 2015; Yang et al., 2020). Consistent with previous studies (He et al., 2012; Yang et al., 2013), many genes involved in the heat shock response were significantly upregulated in response to ethanol stress in ZM4, such as *grpE* (*ZMO0016*), *groES* (*ZM O 1928*), and *groEL* (*ZM O 1929*) (Supplementary Table S3). Further analysis discovered that more general stress response genes were induced by ethanol in ZM4-Δ*hfq*, including 11 genes involved in protein remodeling and reactivation, six genes involved in DNA replication and repair, and three genes responsive for oxidative stress. This was in accordance with the cell growth performance that ZM4-Δ*hfq* was more sensitive to ethanol stress (Figure 1). However, most of these stress response genes were significantly repressed by ethanol in strain ZM4-*hfq*.

Moreover, the gene expression in the strain comparisons showed that these stress response genes, especially genes encoding the heat shock protein (HSP) including *grpE* (*ZMO0016*), *dnaK* (*ZMO0660*), *dnaJ* (*ZMO0661*), *groES* (*ZMO1928*), and *groEL* (*ZMO1929*) were significantly downregulated in ZM4-*hfq* compared with the wild-type ZM4 or ZM4-Δ*hfq* under 8% ethanol (Table 1; Supplementary Table S4). These results collectively suggested that the stress responses induced by ethanol might be effectively alleviated by *hfq* overexpression in ZM4-*hfq*. Therefore, the strain did not have to upregulate its energy-costly protein repair system to conserve energy for ethanol stress responses and cell growth.

In many bacteria, RpoH (Sigma 32) is associated with the stress responses especially the heat shock proteins (HSPs) (Krajewski et al., 2014; Roncarati and Scarlato, 2017). Corresponding to the upregulation of HSPs, the expression of *rpoH* (*ZMO0749*) was induced two folds in ZM4-Δ*hfq* compared with ZM4 under ethanol treatment (Supplementary Table S4). Interestingly, recent study in *Z. mobilis* reported that *rpoH* overexpression led to a decrease in ethanol tolerance (Benoliel et al., 2020). Such evidence suggested that RpoH as well as HSPs at specific concentrations is required for *Z. mobilis* to respond to ethanol stress, and *rpoH* upregulation could make *Z. mobilis* sensitive to ethanol stress as observed in ZM4-Δ*hfq* (Figure 1).

Different from the upregulation of *rpoH* in ZM4-Δ*hfq*, the expression of *rpoH* was not affected in ZM4-*hfq* compared with ZM4 under ethanol treatment, even though the RpoH-transcribed heat shock regulons were downregulated (Supplementary Table S4). As a result, Hfq was speculated to be involved in the regulation of heat shock response *via* the alternative sigma factor RpoH. Previous studies demonstrated that RpoH activity is modulated *via* the DnaK/J-mediated negative feedback loop (Roncarati and Scarlato, 2017; Yura, 2019). In addition, Hfq was recently discovered to be capable to establish many protein-protein interactions in bacterial species (Yonekura et al., 2013; Dos Santos et al., 2019). Collectively, we speculated that *hfq* was associated with the indirect influence to RpoH activity *via* interaction with DnaK and/or DnaJ to help alleviate the cytoplasmic stress responses in *Z. mobilis* under ethanol stress.

Different methods have been developed to characterize the protein-protein interactions, such as co-immunoprecipitation (Co-IP), bacteria/yeast two-hybrid (B2H/Y2H), affinity-based techniques, and split protein complementation assays (Miura, 2018; Pichlerova and Hanes, 2021). Recently, Yi et al. (2013) developed a TEV protease mediated Yeast Endoplasmic Reticulum Sequestration Screening (YESS) system which can be easily applied in eukaryotic and prokaryotic species to explore the protein-protein interactions. In this system, the intracellular protein-protein interaction is converted to quantitative fluorescence signals through a split TEV protease-mediated proteolytic reaction, which possesses many advantages such as signal amplification, clean background and high efficiency. To illustrate the hypothesis above, the YESS system was established in *Z. mobilis* (Supplementary Figure S1) and then applied to detect the interaction between Hfq and DnaK/J.

**FIGURE 5:**
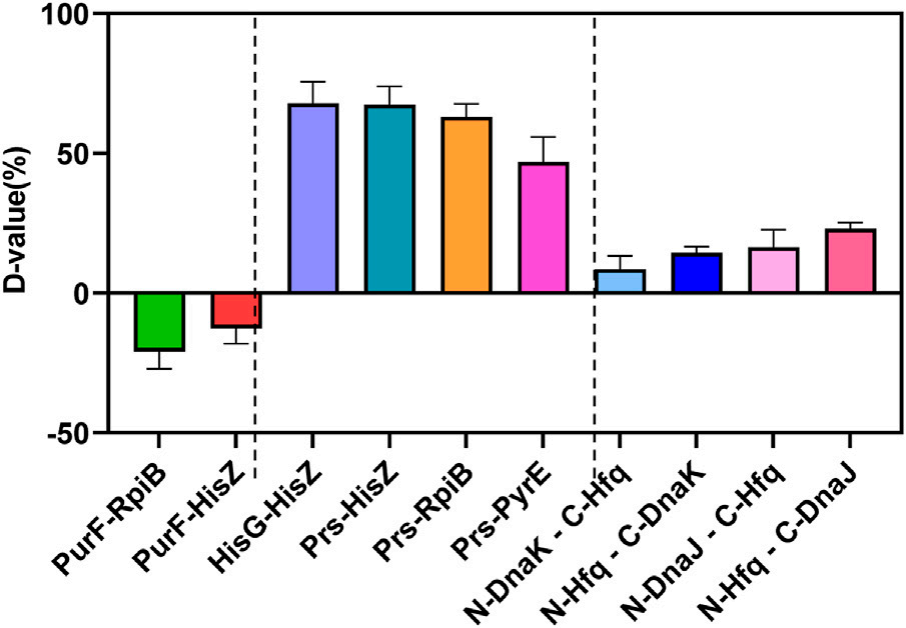
Protein-protein interactions of Hfq-DnaK and Hfq-DnaJ detected by the TEV protease mediated Yeast Endoplasmic Reticulum Sequestration Screening system. D-value: the difference of mono fluorescence signal between experimental samples and control pESE; PurF-RpiB, PurF-HisZ: negative control of protein-protein interaction; HisG-HisZ, Prs-HisZ, Prs-RpiB; Prs-PyrE: positive control of protein-protein interaction.

As shown in Figure 5 and Supplementary Figure S2, the protein-protein interaction in YESS system was quantized by the D-value with flow cytometry. If the D-value >0, the protein pair has interaction, otherwise without interaction. Firstly, several known protein pairs of negative (PurF-RpiB, PurF-HisZ) and positive (HisG-HisZ, Prs-HisZ, Prs-RpiB, Prs-PyrE) ones were selected based on the UniProt and String database, and further evaluated. As expected, all the negative ones (PurF-RpiB, PurF-HisZ) displayed D-value <0, while the positive ones (HisG-HisZ, Prs-HisZ, Prs-RpiB, Prs-PyrE) showed D-value >0 (Figure 5 and Supplementary Figure S2). The results confirmed the YESS system was successfully established *in Z. mobilis.*

The protein pairs of Hfq-DnaK and Hfq-DnaJ were then characterized, which were linked with N-TEV or C-TEV respectively to avoid the structure interference in the protein interaction. Our results showed that the Hfq protein had direct interactions with DnaK and DnaJ in *Z. mobilis*, even though the interaction was not very strong with D-value from 8.45 to 22.98. The data indicated that Hfq was associated with the modulation of RpoH by direct interaction with DnaK/J. Taken together, the regulation of the RpoH-mediated cytoplasmic stress responses by Hfq contributed to the ethanol tolerance in *Z. mobilis*.

## Conclusion

**FIGURE 6:**
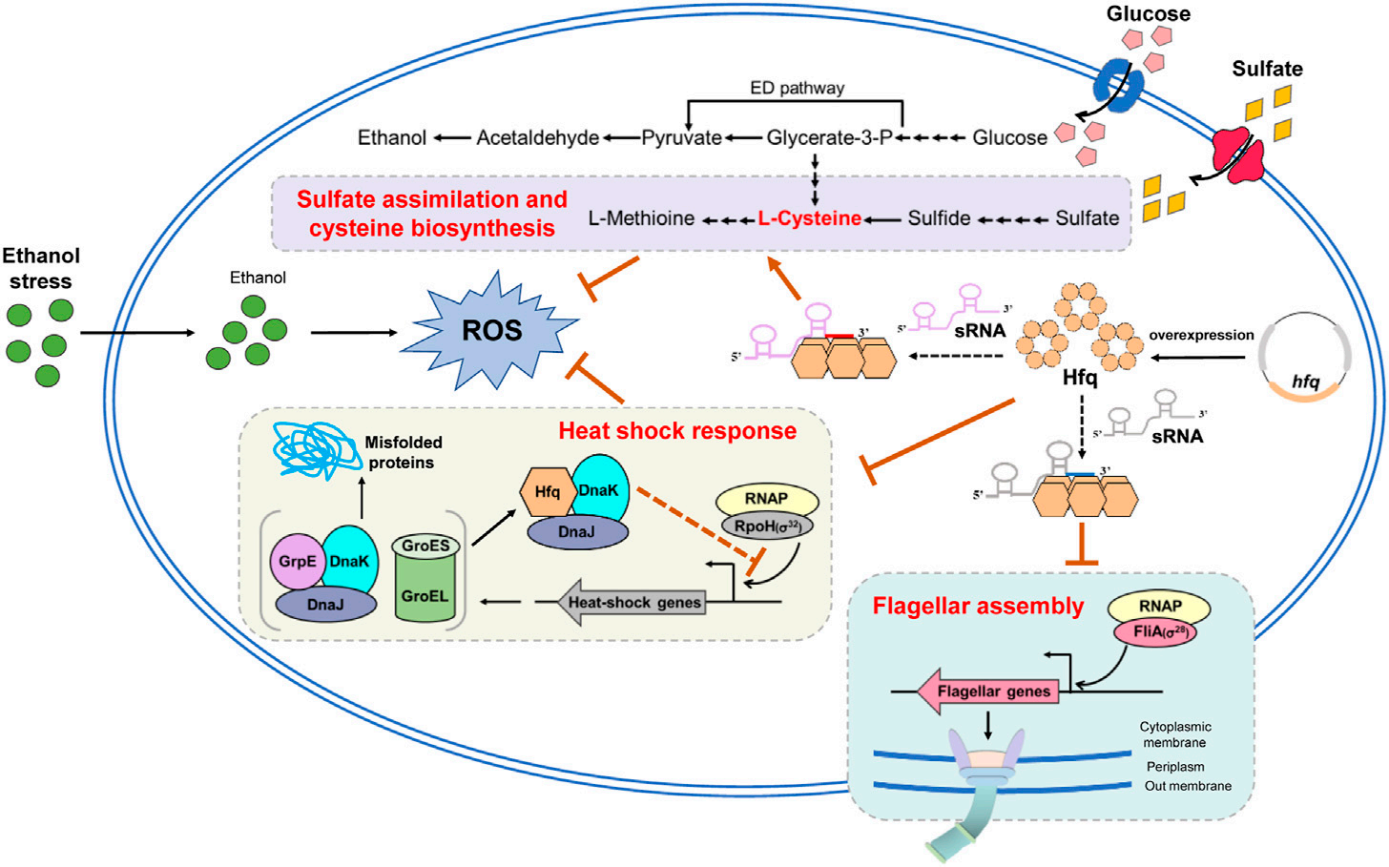
Proposed molecular mechanism of enhanced ethanol tolerance associated with *hfq* overexpression in *Z. mobilis.* Hfq overexpression helps negatively regulate the flagellar related genes and conserve energy for ethanol stress response and cell growth. Moreover, Hfq overexpression enhances sulfate assimilation and cysteine biosynthesis and eliminates the oxidative stress induced by ethanol inhibition. In addition, Hfq indirectly modulates the RpoH through interaction with DnaK/J and appropriately regulates the RpoH-mediated heat shock response under ethanol stress. ED pathway, Entner-Doudoroff pathway; ROS, reactive oxygen species. The orange arrows and orange bars represent upregulate and downregulate, respectively. Solid lines in this figure represent the confirmed regulations and dotted lines represent that are not yet confirmed.

The accumulation of ethanol produced during fermentation is a bottleneck for bioethanol production improvement in *Z. mobilis*. This study exhibited that *hfq* overexpression enhanced ethanol tolerance and increased cell biomass in *Z. mobilis*. Combining transcriptomic analysis, biochemical, and genetics studies, the results identified that the improved ethanol tolerance by *hfq* overexpression is probably due to energy saving by downregulating flagellar biosynthesis and heat shock stress response proteins as well as reducing the ROS induced by ethanol stress *via* upregulating the sulfate assimilation and cysteine biosynthesis (Figure 6). This study gave a promising start for the characterization of the detailed regulatory mechanisms of Hfq in the stress responses in *Z. mobilis*. In addition, the YESS system established in this study provided an alternative approach to investigate the protein-protein interactions in *Z. mobilis*.

## Data Availability

The datasets presented in this study can be found in online repositories. The names of the repository/repositories and accession number(s) can be found in the article/Supplementary Material.
