# Supplementary material for: Molecular mechanism of enhanced ethanol tolerance associated with hfq overexpression in Zymomonas mobilis
Source: Front Bioeng Biotechnol. 2022 Dec 15;10:1098021. doi: 10.3389/fbioe.2022.1098021 (PMC9797736; doi:10.3389/fbioe.2022.1098021)
Supplement: Supplementary file 1 [file DataSheet1.docx]

**Additional file 1**


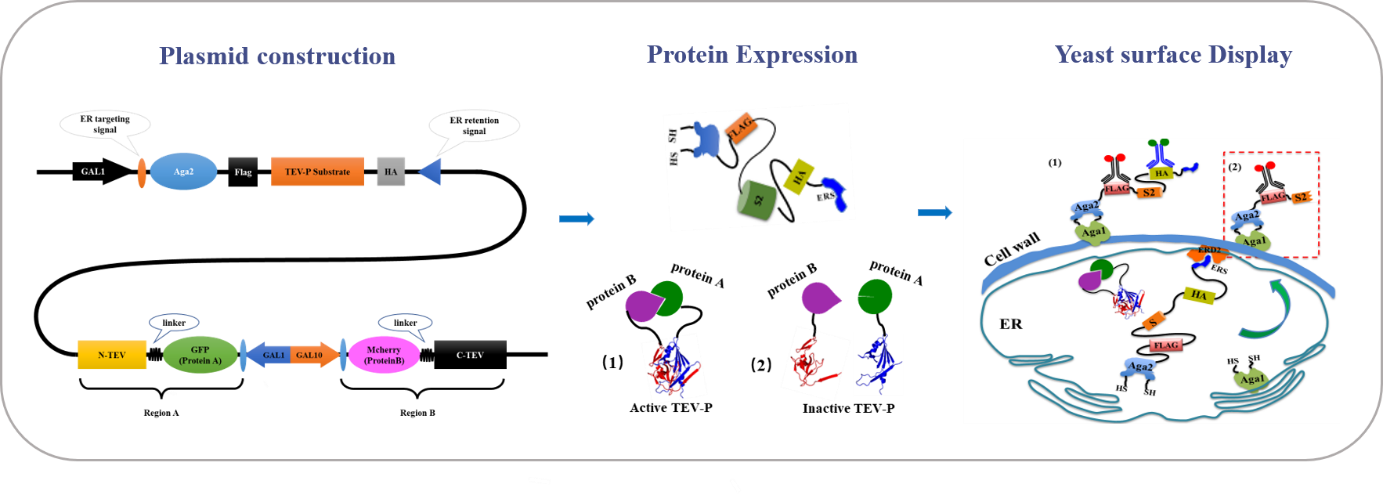


**FIGURE S1** Schematic for the rationale of the YESS system. ERS: ER retention sequence; Aga1 and Aga2: subunits of the yeast adhesion receptor.


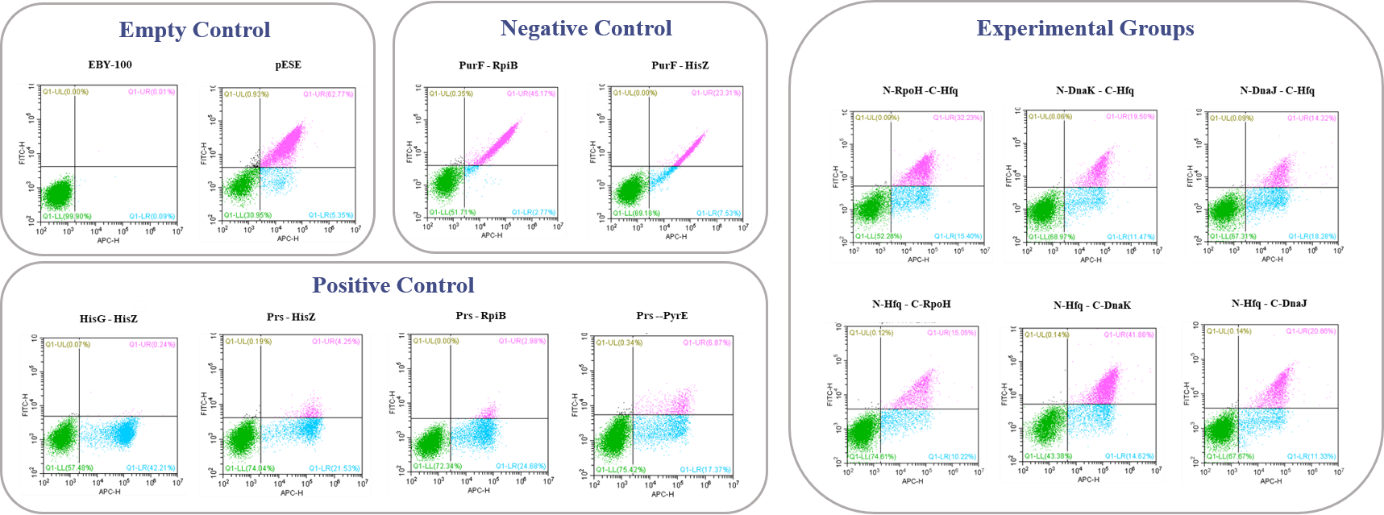


**FIGURE S2**  Representative flow cytometry analysis of displayed fabs on Yeast cell surface (one of three independent experiments). EBY-100: *S. cerevisiae* EBY-100 cells; pESE: *S. cerevisiae* EBY-100 cells transformed pESE (include no protein pairs).

**TABLE S1** Strains and plasmids used in this study.

| **Strains/Plasmids** | **Description** | **Reference** |
| --- | --- | --- |
| **Strains** | | |
| ZM4 | *Z. mobilis* wild‑type strain | Lab stock |
| ZM4-*hfq* | ZM4 containing plasmid pEZ-P*n*-*hfq* | This work |
| ZM4-Δ*hfq* | ZM4 derivative with the entire *hfq* gene deleted and containing plasmid pEZ15Asp | This work |
| DH5α | *E. coli* for plasmid construction | Lab stock |
| Trans110 | *E. coli* for plasmid demethylation | Lab stock |
| EBY100 | *S. cerevisiae*, *MATα ura3-52 trp1 leu2∆1 his3∆200 pep4::HIS3 prb1∆1.6R can1 GAL (pIU211: URA3)* | Lab stock |
| PPI-1 | *S.cerevisiae EBY100* containing plasmid pESE-1 | This work |
| PPI-2 | *S.cerevisiae EBY100* containing plasmid pESE-2 | This work |
| PPI-3 | *S.cerevisiae EBY100* containing plasmid pESE-3 | This work |
| PPI-4 | *S.cerevisiae EBY100* containing plasmid pESE-4 | This work |
| PPI-5 | *S.cerevisiae EBY100* containing plasmid pESE-5 | This work |
| PPI-6 | *S.cerevisiae EBY100* containing plasmid pESE-6 | This work |
| PPI-7 | *S.cerevisiae EBY100* containing plasmid pESE-7 | This work |
| PPI-8 | *S.cerevisiae EBY100* containing plasmid pESE-8 | This work |
| PPI-9 | *S.cerevisiae EBY100* containing plasmid pESE-9 | This work |
| PPI-10 | *S.cerevisiae EBY100* containing plasmid pESE-10 | This work |
| **Plasmids** | | |
| pEZ15Asp | Shuttle vector contains *Z. mobilis* origin and *E. coli* origin p15A; Sp^R^; Biobrick-compatible | Yang et al., 2016 |
| pEZ-P*n*-*hfq* | pEZ containing construct Pn*-hfq*: codon- *hfq* gene driven by native promoter P*native* | This work |
| pL2R | pEZ15Asp containing a DNA fragment of two tandem copies of CRISPR repeat; for artificial CRISPR loci construction | Zheng et al., 2019 |
| pKO-*hfq* | pL2R derivative containing a CRISPR locus with a spacer matching a protospacer in *hfq*, and a donor of recombination arms homologous to the sequences flanking *hfq* | This work |
| pESD | Basic vector for yeastcell surface display | Yi et al., 2013 |
| pESE | Vector modified from pESD containing fragments of *GAL1-gfp-GAL10-mCherry*, CEN, TEV protease, ENLYQGS,*Trp-* | This work |
| pESE-1 | Vector modified from pESE containing fragments of *GAL1-purF-GAL10-rpiB*, CEN, TEV protease, ENLYQGS,*Trp-* | This work |
| pESE-2 | Vector modified from pESE containing fragments of *GAL1-purF-GAL10-hisZ*, CEN, TEV protease, ENLYQGS,*Trp-* | This work |
| pESE-3 | Vector modified from pESE containing fragments of *GAL1-hisG-GAL10-hisZ*, CEN, TEV protease, ENLYQGS,*Trp-* | This work |
| pESE-4 | Vector modified from pESE containing fragments of *GAL1-prs-GAL10-hisZ*, CEN, TEV protease, ENLYQGS,*Trp-* | This work |
| pESE-5 | Vector modified from pESE containing fragments of *GAL1-prs-GAL10-rpiB*, CEN, TEV protease, ENLYQGS,*Trp-* | This work |
| pESE-6 | Vector modified from pESE containing fragments of *GAL1-prs-GAL10-pyrE*, CEN, TEV protease, ENLYQGS,*Trp-* | This work |
| pESE-7 | Vector modified from pESE containing fragments of *GAL1-dnaK-GAL10-hfq*, CEN, TEV protease, ENLYQGS,*Trp-* | This work |
| pESE-8 | Vector modified from pESE containing fragments of *GAL1-hfq-GAL10-dnaK*, CEN, TEV protease, ENLYQGS,*Trp-* | This work |
| pESE-9 | Vector modified from pESE containing fragments of *GAL1-dnaJ-GAL10-hfq*, CEN, TEV protease, ENLYQGS,*Trp-* | This work |
| pESE-10 | Vector modified from pESE containing fragments of *GAL1-hfq-GAL10-dnaJ*, CEN, TEV protease, ENLYQGS,*Trp-* | This work |

**TABLE S2** Primers used in this study.

| **Primer** | **Sequence (5'-3')** |
| --- | --- |
| Pn-*hfq*-F | tcaccgtctgaattcaggcttggatgctggttttc |
| pEZ-Pn-*hfq*-R | gccgctactagtttaatcctcgtctcgcctttctgtc |
| *hfq*-guide RNA-F | gaaagtaatgacgacgcgcagctggtttataaacat |
| *hfq*-guide RNA-R | gaacatgtttataaaccagctgcgcgtcgtcattac |
| *hfq*-CRISPR-upstream-F | ggtcaccagctcaccgtctgtttggctttaacgcaggttcag |
| *hfq*-CRISPR-upstream-R | gaccttttcggccattgtcc |
| *hfq*-CRISPR-downstream-F | ggacaatggccgaaaaggtccgagacgaggattgagtgg |
| *hfq*-CRISPR-downstream-R | tcgagagatcgatatcactggaatagcaaattccccgcc |

**References**

Yang, S., Mohagheghi, A., Franden, M. A., Chou, Y. C., Chen, X., Dowe, N., Himmel, M. E., & Zhang, M. (2016). Metabolic engineering of *Zymomonas mobilis* for 2,3-butanediol production from lignocellulosic biomass sugars. *Biotechnology for Biofuels*, 9(1), 189.

Zheng, Y., Han, J., Wang, B., Hu, X., Li, R., Shen, W., Ma, X., Ma, L., Yi, L., Yang, S., & Peng, W. (2019). Characterization and repurposing of the endogenous Type I-F CRISPR-Cas system of *Zymomonas mobilis* for genome engineering. *Nucleic Acids Research,* 47(21), 11461–11475.
